# The effect of bone marrow-derived stem cells associated with platelet-rich plasma on the osseointegration of immediately placed implants

**DOI:** 10.4317/jced.56743

**Published:** 2021-01-01

**Authors:** Roberta-Targa Stramandinoli-Zanicotti, Laurindo-Moacir Sassi, Carmen-Lucia-Kuniyoshi Rebelatto, Lidiane M. Boldrine-Leite, Paulo-Roberto Brofman, Andre-Lopes Carvalho

**Affiliations:** 1 Postgraduate Program in Oncology, University of São Paulo, São Paulo, SP, Brazil; Oral and Maxillofacial Surgery Department, Erasto Gaertner Hospital, Curitiba, PR, Brazil; 2 Oral and Maxillofacial Surgery Department, Erasto Gaertner Hospital, Curitiba, PR, Brazil; 3 Experimental Laboratory of Cell Culture, Pontifical Catholic University of Paraná (PUC-PR), Curitiba, PR, Brazil; 4 Research Advisor of Postgraduate Program in Oncology, Medical School of University of São Paulo (FMUSP), São Paulo, SP, Brazil

## Abstract

**Background:**

Stem cells associated with growth factors have been shown to improve bone healing and the osseointegration of dental implants. A Brazilian miniature pig model was used to evaluate the effect of autologous bone marrow-derived mesenchymal stem cells (BM-MSCs) associated with platelet-rich plasma (PRP) on the osseointegration of immediately placed dental implants.

**Material and Methods:**

A total of four male adult miniature pigs were used in this study. BM-MSCs from each pig were isolated from the iliac crest and expanded *in vitro*. The undifferentiated BM-MSCs were mixed with autologous PRP and implanted in the post-extraction sockets at the experimental sites before implant placement (10 x 106 cells/ socket). The control sites did not receive either BM-MSC or PRP. Each animal received four implants in the control side and 04 on the experimental side, totalizing 32 implants. The specimens were analyzed radiographically and histomorphometrically to determine the implant loss rate (ILR), the bone-implant contact (BIC), and bone density within the threads (BDWT).

**Results:**

The ILR, the BIC, and the BDWT for the control and experimental sites were respectively 25.0% and 18.7% (*p*=0.686); 39.0% and 27.7% (*p*=0.110); 46.8% and 36.5% (*p*=0.247).

**Conclusions:**

The use of BM-MSCs + PRP in conjunction with immediately placed implants showed a lower ILR but there was no significant effect on the osseointegration of the dental implants. More preclinical studies, in large animal models, are needed to establish whether BM-MSCs associated with PRP could be used for the enhancement of the osseointegration of dental implants.

** Key words:**Osseointegration, bone marrow-derived mesenchymal stem cells, platelet-rich plasma, dental implants, minipigs.

## Introduction

Dental implant therapy is a well-accepted treatment modality to replace missing teeth (1). The maintenance of osseointegration with minimal bone loss over time indicates the success of dental implants (2,3). Insufficient amounts of bone present during the installation of dental implants is a common finding, especially in the case of immediate dental implant placement (4).

It is well known that autogenous bone is gold standard for bone augmentation since autogenous grafts have unique characteristics such as osteogenesis, osteoinductivity, and osteoconductivity (5). However, its clinical applications are very limited due to extra surgical time, donor site morbidity, high resorption rate, and in some cases limited availability (6). Stem cell therapy has emerged as an alternative to bone grafting since it has been applied to promote bone regeneration and improve the conditions of the osseointegration of dental implants (7).

Numerous pre-clinical studies have shown that stem cells can promote bone regeneration and aid the integration of dental implants. However, no solid conclusion can be drawn from the very limited number of clinical studies conducted so far (8).

Mesenchymal stem cells (MSC) are non-hematopoietic progenitor cells that can differentiate into distinct mesenchymal cell lineages, including osteoblastic, condroblastic and adipogenic lineages (9). MSCs isolated from bone marrow (BM-MSCs) have been used in regenerative medicine (10), including bone tissue engineering, offering a hopeful opportunity to repair peri-implant bone defects (7). The use of MSC can be an alternative to classic tissue regeneration techniques, offering beneficial effects in bone healing around dental implants (11).

Platelet-rich plasma (PRP) is a concentrate of platelets obtained from whole autologous blood following centrifugation. In the dental field, autologous PRP is commonly used combined with dental implants and bone graft, to promote soft and hard tissue healing (12). A number of experimental studies have shown that the use of MSCs associated with growth factors such as PRP improves bone healing and osseointegration (4,7,13-16).

We used an animal model to evaluate the effect of BM-MSCs associated with autologous PRP (BM-MSCs + PRP) on the osseointegration of dental implants immediately placed in post-extraction sites in the mandibles of miniature pigs. We hypothesized that the osteogenic effect of this association will lead to improved bone formation and apposition on the surface of the implants, showing a positive effect on osseointegration.

## Material and Methods

-Animals and groups

This study included four 18 months old male Brazilian miniature pigs (BR1; Minipig Pesquisa & Desenvolvimento LTDA, São Paulo, Brazil) weighting 40-50 kg. This study was approved by the Ethics and Research Committee of the Positivo University, Curitiba, PR, Brazil (Protocol# 001/2009) and followed the National Institute of Health guidelines for the care and use of experimental animals.

-Isolation, expansion, and culture of BM-MSCs 

All animals underwent the same procedures. About 20 mL of fresh heparinized BM was aspirated from the posterior iliac crest using a Jamishidi needle (Raiomedic, São Jose dos Pinhais, PR, Brazil). Mononuclear cells were isolated by Ficoll-Hypaque (Sigma-Aldrich Corp, St. Louis, MO, USA) density gradient centrifugation (d = 1,077 g/cm3), and MSCs were selected by plastic-adherence (17). At passage four, the immunophenotypic profile was determined by flow cytometry, and *in vitro* differentiation was performed. Cell morphology assessment, surface antigens expression, and cellular differentiation into adipocytes, osteoblasts, and chondroblasts were performed to confirm the BM-MSCs phenotype as previously described (18).

-Preparation of PRP 

Platelets were obtained from the venous blood of each animal. Blood was drawn from the auricular vein into a sterile microtube containing sodium citrate anticoagulant 3.2% (Becton Dickinson, NJ, USA). A total of 30mL of blood was centrifuged twice: first at 130x g for 10min at 22ºC to remove red blood cells, and a second time at 400x g for 10 min at 22ºC to obtain PRP containing 1x106 platelets/ml. Red blood cells precipitate in the tube bottom and the upper part forms a “fog zone” composed of a few erythrocytes, monocytes, and platelets higher. The upper part containing poor plasma and platelet-rich plasma was pipetted along with 1 mL of the mist zone and replaced in another conical tube and centrifuged again at 400x g for 10 minutes at 22ºC. After the second centrifugation of the plasma, 50% was collected and packaged in another tube constituting plasma poor platelet. This was used for the formation of the autologous thrombin, while the remaining material was resuspended constituting PRP. The platelet concentrate was activated by adding calcium gluconate (100mg/ml) and autologous thrombin. The PRP was immediately used on the experimental sites of each animal.

-Surgical Procedure 

**Figure 1:**
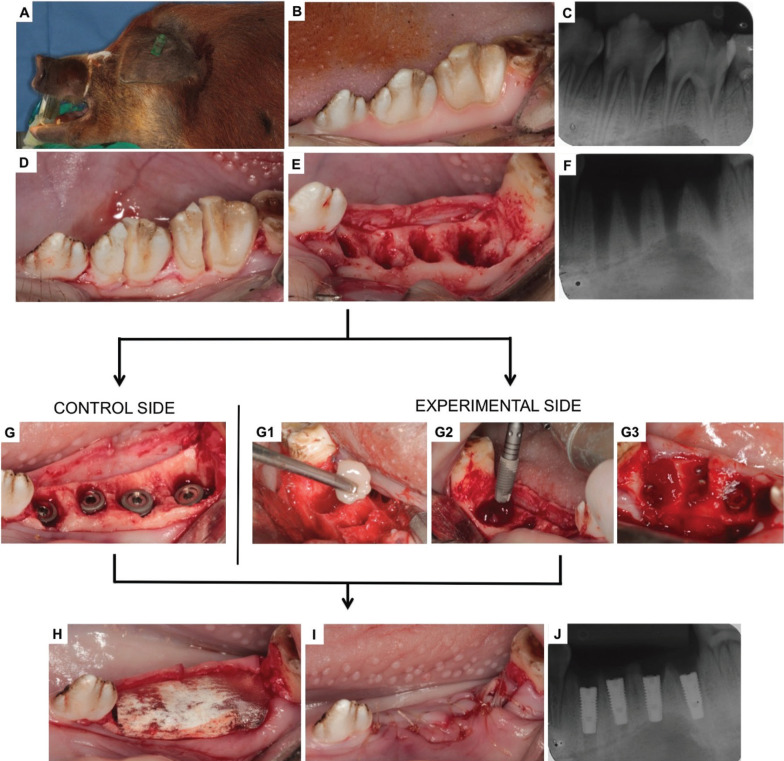
Surgical procedures sequence: A) Animal under general anesthesia; B) Initial clinical aspect of the premolar teeth; C) Initial radiographic features; D) Sagittal section; E) Fresh alveolus; F) Post-extraction radiographic features; G) Immediately-placed implants on the control side; G1) BM-MSCs+PRP gel; G2-G3) Implants + BM-MSCs+PRP on the experimental side; H) Placement of the collagen membrane; F) Suture and J) Final radiographic features.

Pre-medication consisted of intramuscular 10 − 20 mg/kg ketamine, followed by an intravenous bolus of 10 mg midazolam. Continuous sedation was maintained with intravenous 0.2–1.0 mg/kg/hr midazolam and 0.1–0.2 mcg/kg/hr fentanyl. Intraoral antisepsis was done with a 0.12% chlorhexidine solution. The third and the fourth premolars on each side of the mandible were extracted after longitudinal root sectioning. Each animal was used either for the test or control sites. On the control side, four titanium implants with 3.5x11mm (ConeMorse; Neodent, Curitiba, Brazil) were placed in the fresh extraction sockets. At the experimental side, before the placement of the implants, BM-MSCs in culture (undifferentiated cells in passage four) were trypsinized, centrifuged, pelleted, and mixed with autologous PRP. The mixture PRP/BM-MSC was used to fill the socket (10 x 106 cells/socket) before the implant placement. For protection, a resorbable bovine membrane (GenDerm/ Baumer, Sao Paulo, Brazil) was applied to all sites for both test and control. The gingival flaps were sutured using interrupted sutures with Vicryl Rapid 2.0 (Ethicon, São Jose dos Campos, Brazil). A total of 32 implants were placed equally divided between test and control. The surgical procedure sequence is illustrated in Figure 1.

The postoperative care protocol was intravenous 3.0 mg/kg morphine and 7.5 mL kinetomax. After each surgery, the animals were fed only with food powder (Presuntina Pró, Purina, Brazil), and no specific oral hygiene procedures were performed. The miniature pigs were euthanized at 90 days after the surgeries. The hemi-mandibular bone was dissected and removed from each animal. X-rays of the bone blocks were taken and used for determining the implant lost rate (ILR) for each side.

-Histological Processing 

The bone blocks containing the dental implants were fixed in 10% buffered formalin for two weeks and then embedded in acrylic resin. After complete polymerization, the acrylic blocks were sectioned parallel to the long axis of the implant, using a cutting machine (EXAKT-Cutting Grinding System 400CP; Kulzer, Norderstedt, Germany).

One side of the cut was then polished with non-ferrous silicon carbide abrasive files, with different granulations, used in a polishing machine (Politriz Petalográfica DP-10, Panambra / Struers, Cambuci, SP, Brazil), under constant water irrigation. After polishing, the piece was dried with absorbent paper and the polished side glued with cyanoacrylate (Super Bonder, 3M) on a 2 mm thick colorless and transparent acrylic sheet for handling and final finishing. The other side of the cut was then polished in the same sanding sequence, to an average final thickness of 10 μm, measured using a digital caliper (Mitutoyo, Brazil). For histological and histomorphometric analyses, sections were stained with Toluidine Blue.

-Histological Evaluation

The samples underwent histomorphometric analysis for the percentage of the bone-implant contact (BIC) and the bone density within the threads (BDWT) using ImageJ (NIH, USA). The measurements were obtained from the entire length of the implants on both sides, and then the average was calculated. The percentage of BIC was calculated by evaluating the total amount of mineralized bone in contact with both sides of the surface of the implant, multiplying by 100, and divided by the total linear measure of the implant. The percentage of the BDWT was calculated by analyzing the area of the newly formed bone within the areas formed inside all threads of the implant, multiplying by 100 and divided by the total area.

-Statistical Analysis 

Continuous variables are presented as average, median, maximum, minimum, and standard deviations. Categorical variables are presented as frequencies and percentages. The comparison between control and experimental sides was performed by using the non-parametric Mann-Whitney U test. Significance was established at p <  0.05. Analyses were performed with SPSS statistics software (V.14 IBM Corporation, New York, USA).

## Results

Of the total implants installed, four were lost on the control site and three on the experimental site. The control side presented a higher ILR but this difference was not statistically significant (*p*=0.686). The number of implants lost and the ILR are demonstrated in Table 1.

**Table 1:**
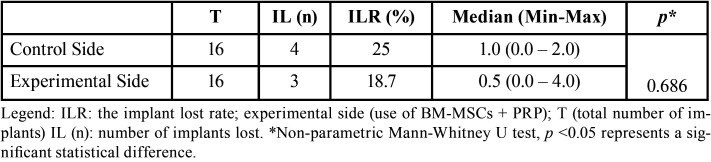
Descriptive statistics of ILR.

One sample on the experimental side did not show any amount of mineralized bone tissue in contact with the implant surface and was considered fibrointegrated. The BIC and the BDWT for the control and experimental sites were respectively 39.0% and 27.7%, 46.8% and 36.5% (*p*=0.247). There was no significant statistical difference between the sides in both criteria (*p*=0.110 and *p*=0.247 respectively). The BIC and the BDWT in the control and experimental sides are demonstrated in Table 2.

**Table 2:**
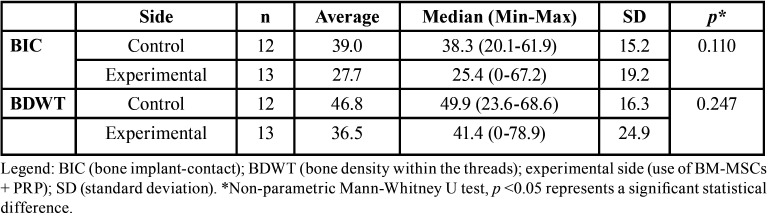
Descriptive statistics of BIC and BDWT.

**Figure 2:**
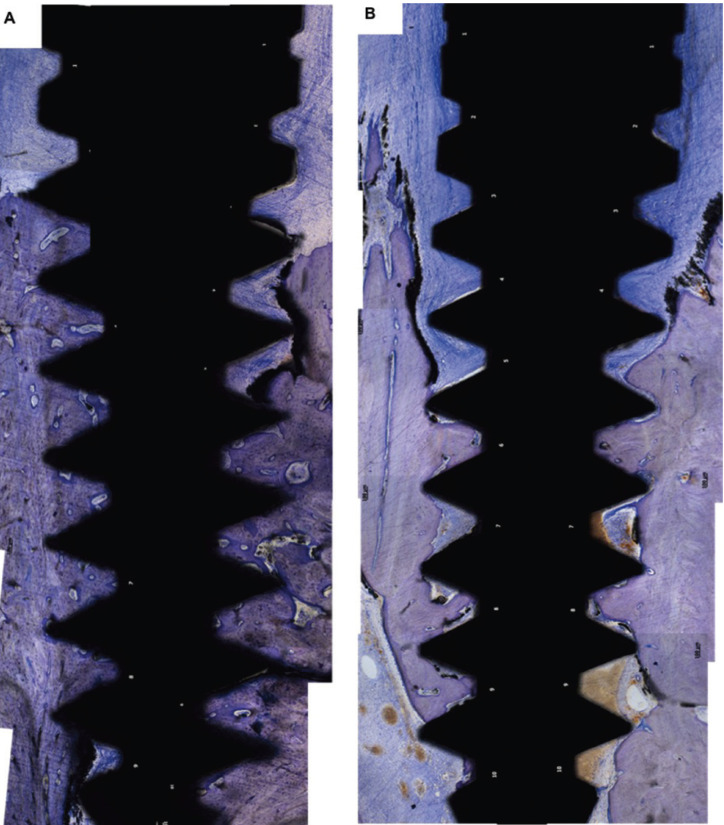
Histological features of samples of control (A) and experimental (B) sides (Stain: Toluidine Blue; Bar:100μm.

Histological features of the control and experimental side can be found in Figure 2.

## Discussion

Experimental studies in animals allow the translation of the results into the clinical practice and the development of novel strategies for regenerative therapies aiming to accelerate the process of bone neoformation and osseointegration around dental implants. *In vivo* studies are able to evaluate the biocompatibility and the regenerative capacity of biomaterials (11).

Dental experiments in animal models such as miniature pig, dogs, and sheep, goats are difficult to perform for several reasons, such as animal size, the cost, and availability, difficulty in maintenance, feeding, and postoperative care. The early loss of dental implants installed in mandible or maxilla in animal models is commonly described in the literature, since it is not possible to maintain the postoperative care necessary for the implant osseointegration, such as pasty / liquid feeding, avoiding trauma in the operated area, and the use of topical antimicrobials such as chlorhexidine. For these reasons, several researchers evaluate the osseointegration of dental implants in other bones such as the tibia or skullcap. Despite its limitations, the installation of the implants in the jawbone makes the chosen surgical model the closest to the clinical reality. When a dental implant is installed in a bone covered by muscle and skin, the process of peri-implant bone formation is different from that installed in the mandible or maxilla, since it does not receive chewing trauma, and also there is no interaction with oral bacteria. Also, the choice of model and methods are related to the question being answered and not necessarily whether it correlates with clinical scenarios.

A number of authors have demonstrated the positive effect of the association of BM-MSCs+PRP on bone formation and bone regeneration (13-15,19-21). A recent experimental study in dogs demonstrated that MSC treatment could be very useful for bone repair and dental implant osseointegration (4). In contrast, our study demonstrated that there was no statistically significant difference between experimental and control side, even though the number of implants lost was lower on the experimental side. In the same way, the percentages of BIC and BDWT were worse on the experimental side in relation to the control side although not statistically significant. This indicated, in contrast with others, that the use of BM-MSCs+PRP did not show a positive effect in any of the parameters analyzed (15,21). The presence of leukocytes in the platelet concentrate interferes directly with the PRP properties (22). Leukocytes play an essential role in the early stages of inflammation and are also responsible for increasing the potential of growth factors (23), which may lead to an unfavorable repair response of the tissues such as fibrosis and bone formation inhibition (24). Similar to our study, previous research did not find a positive effect of PRP on bone regeneration (7,26,27). The effects of PRP are likely to be limited by the quick, non-sustained release of growth factors and lack of BMP-2 (25). Moreover, previous studies reported that the adjunctive use of PRP failed to increase bone density and BIC values in peri-implant defects treated with undifferentiated BM-MSCs (7).

The different growth factors released by platelets present in PRP have a significant impact on the proliferation, regulation, and differentiation of MSCs, with an increase in bone formation capacity (15,19,28-30). However, bone defects treated with PRP showed increased expression of type III and type I collagen, in addition to a decrease in bone mineralization, inducing the granulation tissue and bone marrow, associated with a thrombogenic effect (26,27). In this sense, new investigations must be carried out to clarify the relationship and the signaling pathway of PRP and MSCs on dental implant osseointegration.

## Conclusions

The use of BM-MSCs + PRP along immediately placed implants in fresh post-extraction sockets of miniature pigs showed a non-significant lower implant loss rate. Also, there was no significant positive effect on bone regeneration and osseointegration. More preclinical studies in large animal models are needed to demonstrate whether BM-MSCs associated with growth factors such as PRP can be considered a safe option for aiding bone regeneration and the osseointegration of dental implants.

## References

[1] Mertens C, Meyer-Bäumer A, Kappel H, Hoffmann J, Steveling HG (2012). Use of 8-mm and 9-mm implants in atrophic alveolar ridges: 10-year results. Int J Oral Maxillofac. Implants.

[2] Albrektsson T, Zarb GA, Worthington P (1986). The long-term efficacy of currently used dental implants: A review and proposed criteria of success. Int J Oral Maxillofac Implants.

[3] Brånemark R, Öhrnell LO, Skalak R, Carlsson L, Brånemark P I (1998). Biomechanical characterization of osseointegration: An experimental in vivo investigation in the beagle dog. J Orthop Res.

[4] Bressan E, Botticelli D, Sivolella S, Bengazi F, Guazzo R, Sbricoli L (2015). Adipose-Derived Stem Cells as a Tool for Dental Implant Osseointegration: an Experimental Study in the Dog. Int J Mol Cell Med.

[5] Kao ST, Scott DD (2007). A review of bone substitutes. Oral Maxillofac. Surg. Clin. North Am.

[6] Sjöström M, Sennerby L, Nilson H, Lundgren S (2007). Reconstruction of the atrophic edentulous maxilla with free iliac crest grafts and implants: a 3-year report of a prospective clinical study. Clin Implant Dent Relat Res.

[7] Yun JH, Han SH, Choi SH, Lee MH, Lee SJ, Song SU (2014). Effects of bone marrow-derived mesenchymal stem cells and platelet-rich plasma on bone regeneration for osseointegration of dental implants: preliminary study in canine three-wall intrabony defect. J Biomed Mater Res B Appl Biomater.

[8] Viña JA, El-Alami M, Gambini J, Borras C, Viña J, Peñarrocha MA (2014). Application of mesenchymal stem cells in bone regenerative procedures in oral implantology. A literature review. J Clin Exp Dent.

[9] Dominici M, Le Blanc K, Muller E, Slaper-Cortenbach I, Marini F, Krause D (2006). Minimal criteria for defining multipotent mesenchymal stromal cells. The international society for cellular therapy position statement. Cytotherapy.

[10] Strauer BE, Kornowski R (2003). Stem cell therapy in perspective. Circulation.

[11] Misawa MYO, Huynh-Ba G, Villar GM, Villar CC (2006). Efficacy of stem cells on the healing of peri-implant defects: systematic review of preclinical studies. Clinical and Experimental Dental Research.

[12] Marx RE (2004). Platelet-rich plasma: evidence to support its use. J Oral Maxillofac Surg.

[13] Ito K, Yamada Y, Naiki T, Ueda M (2006). Simultaneous implant placement and bone regeneration around dental implants using tissue-engineered bone with fibrin glue, mesenchymal stem cells and platelet-rich plasma. Clin Oral Implants Res.

[14] Pieri F, Lucarelli E, Corinaldesi G, Iezzi G, Piattelli A, Giardino R (2008). Mesenchymal stem cells and platelet-rich plasma enhance bone formation in sinus grafting: a histomorphometric study in minipigs. J Clin Periodontol.

[15] Yamada Y, Nakamura S, Ito K, Kohgo T, Hibi H, Nagasaka T (2008). Injectable tissue- engineered bone using autogenous bone marrow-derived stromal cells for maxillary sinus augmentation: clinical application report from a 2-6-year follow-up. Tissue Eng Part A.

[16] Pieri F, Lucarelli E, Corinaldesi G, Fini M, Aldini NN, Giardino R (2009). Effect of mesenchymal stem cells and platelet-rich plasma on the healing of standardized bone defects in the alveolar ridge: a comparative histomorphometric study in minipigs. J Oral Maxillofac Surg.

[17] Friedenstein AJ, Chailakhjan RK, Lalykina KS (1970). The development of fibro-blast colonies in monolayer cultures of guinea-pig bone marrow and spleen cells. Cell and Tissue Kinetics.

[18] Stramandinoli-Zanicotti RT, Carvalho AL, Rebelatto CL, Sassi LM, Torres MF, Senegaglia AC (2014). Brazilian minipig as a large-animal model for basic research and stem cell-based tissue engineering. Characterization and in vitro differentiation of bone marrow-derived mesenchymal stem cells. J Appl Oral Sci.

[19] Niemeyer P, Schönberger TS, Hahn J, Kasten P, Fellenberg J, Suedkamp N (2009). Xenogenic transplantation of human mesenchymal stem cells in a critical size defect of the sheep tibia for bone regeneration. Tissue engineering: Part A.

[20] Zhang L, Wang P, Mei S, Li C, Cai C, Ding Y (2012). In vivo alveolar bone regeneration by bone marrow stem cells/fibrin glue composition. Archives of Oral Biology.

[21] Man Y, Wang P, Guo Y, Xiang L, Yang Y, Qu Y (2012). Angiogenic and osteogenic potential of platelet-rich plasma and adipose-derived stem cell laden alginate microspheres. Biomaterials.

[22] Ehrenfest DM, Rasmuson L, Albrektsson T (2009). Classification of platelet concentrates: From pure platelet-rich plasma (P-PRP) to leucocyte- and platelet- rich fibrin (LPRF). Trends Biotechnol.

[23] Ehnert S, Baur J, Schmitt A, Neumaier M, Lucke M, Dooley S (2010). TGF-b1 as possible link between loss of bone mineral density and chronic inflammation. PLoS One.

[24] Arnoczky SP, Delos D, Rodeo SA (2011). What is platelet-rich plasma?. Operat Tech Sports Med.

[25] Kumar RV, Shubhashini N (2013). Platelet rich fibrin: a new paradigm in periodontal regeneration. Cell tissue bank.

[26] Giovanini AF, Deliberador TM, Gonzaga CC, de Oliveira Filho MA, Gohringer I, Kuczera J (2010). Platelet-rich plasma diminishes calvarial bone repair associated with alterations in collagen matrix composition and elevated CD34" cell prevalence. Bone.

[27] Giovanini AF, Gonzaga CC, Zielak JC, Deliberador TM, Kuczera J, Goringher I (2011). Platelet-rich plasma (PRP) impairs the craniofacial bone repair associated with its elevated TGF-beta levels and modulates the co-expression between collagen III and alpha-smooth muscle actin. J Orthop Res.

[28] Lynch SE, de Castilla GR, Williams RC, Kiritsy CP, Howell TH, Reddy MS (1991). The effects of short-term application of a combination of platelet-derived and insulin-like growth factors on periodontal wound healing. J Periodontol.

[29] Marx RE, Carlson ER, Eichstaedt RM, Schimmele SR, Strauss JE, Giorgiff KR (1998). Platelet-rich plasma. Growth factor enhancement for bone grafts. Oral Surg Oral Med Oral Pathol Oral Radiol Endod.

[30] Gruber R, Karreth F, Kandler B, Fuerst G, Rot A, Fischer MB (2004). Platelet-released supernatants increase migration and proliferation, and decrease osteogenic differentiation of bone marrow-derived mesenchymal progenitor cells under in vitro conditions. Platelets.

